# Identification of a novel *TGFB1* variant in a patient with Camurati-Engelmann disease responsive to alendronate

**DOI:** 10.1093/rheumatology/keaf441

**Published:** 2025-08-14

**Authors:** Neelam Hassan, Celia L Gregson, Michael Oldridge, Tracy Lester, Emma L Duncan, Jonathan H Tobias

**Affiliations:** Musculoskeletal Research Unit, University of Bristol, Bristol, UK; Department of Rheumatology, Southmead Hospital, North Bristol NHS Trust, Bristol, UK; Musculoskeletal Research Unit, University of Bristol, Bristol, UK; Oxford Regional Genetics Laboratories, Oxford University Hospitals NHS Foundation Trust, Oxford, UK; Oxford Regional Genetics Laboratories, Oxford University Hospitals NHS Foundation Trust, Oxford, UK; Department of Twin Research and Genetic Epidemiology, School of Life Course & Population Sciences, Faculty of Life Sciences and Medicine, King’s College London, London, UK; Musculoskeletal Research Unit, University of Bristol, Bristol, UK; Department of Rheumatology, Southmead Hospital, North Bristol NHS Trust, Bristol, UK

Rheumatology key message

- Whole genome sequencing and alendronate may be of value in diagnosing and managing CED, respectively.

Dear Editor, Camurati-Engelmann disease (CED) is a rare autosomal dominant progressive sclerosing dysplasia characterized by cortical hyperostosis of the long bones and skull [1]. Affected individuals present with bone pain, proximal muscle weakness and easy fatigability. Headaches, cranial nerve impingement and both conductive and sensorineural hearing loss can occur from skull base involvement. CED type 1 is caused by heterozygous activating mutations in *Transforming Growth Factor-Beta-1* (*TGFB1*). Here, we report an individual with a delayed diagnosis of CED and a novel *TGFB1* variant.

A 34-year-old male presented in 2014 with widespread limb and back pain, arthralgia and muscle weakness since adolescence. He had fractured various bones after falls (aged 2, 7, 15 and 21 years). He developed bilateral sensorineural hearing loss in his 20s. He previously had a vitamin D deficiency but reported no symptomatic improvement with supplementation. There was no relevant family history, and he was not taking any regular medication.

Examination showed a slim man (height 173 cm; weight 53.8 kg; BMI 18 kg/m^2^) wearing hearing aids. He had reduced proximal (but not distal) muscle volume in his lower limbs and shortened great toes bilaterally. Examination was otherwise unremarkable.

Investigations showed mildly elevated ALP (155 U/l [reference range [RR] 20–120 U/l]) but normal adjusted calcium (2.47 mmol/l), phosphate (1.29 mmol/l), parathyroid hormone (3.1 pmol/l) and vitamin D (146 nmol/l). Creatinine was low (53 µmol/l) and creatine kinase was normal (37 U/l). Gonadal hormones were unmeasured. Bone turnover markers were markedly elevated (procollagen 1N propeptide [P1NP] 179 µg/l [RR 20–76 µg/l]; serum cross-linked C-telopeptide of type I collagen [CTX] 1.7 µg/l [RR 0.1–0.5 µg/l]). Hand, spine, hip, knee and foot radiographs demonstrated diffuse coarsened sclerotic bones with marked diaphyseal bone expansion (Fig. 1A, B), and Morton’s toes (normal variant affecting 15–20% of the population). DXA showed increased BMD (*Z* scores at lumbar spine (L1-4) +4.1, total hip +3.0, and femoral neck +5.3). Muscle biopsy was inconclusive.

**Figure 1.**
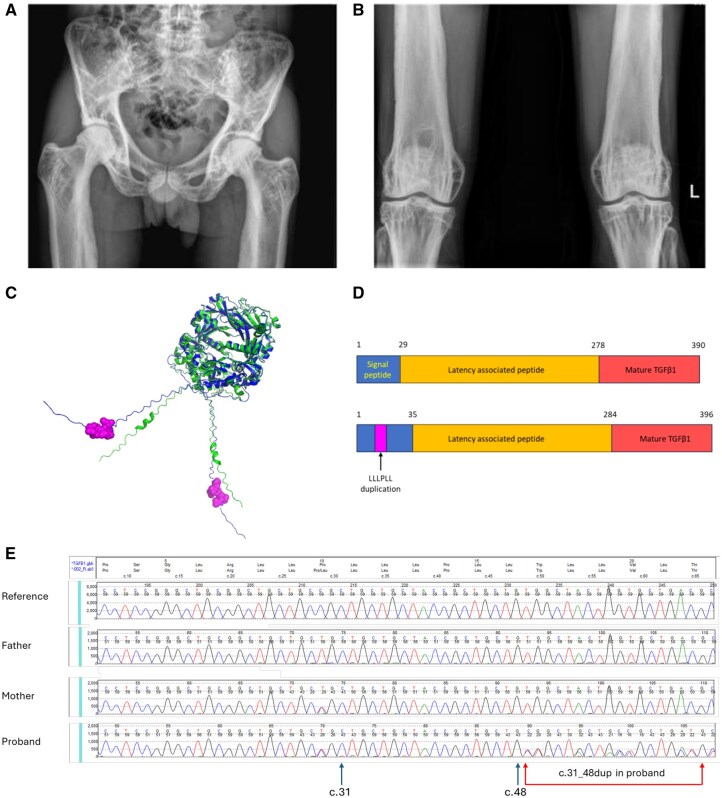
Radiographic, structural and genetic characterization of a novel *TGFB1* variant in a heterozygous carrier. Diffuse coarse trabecular pattern with cortical bone expansion on pelvic (**A**) and knee (**B**) radiographs. (**C**) Protein model of the wild-type pre-pro-TGFβ1 homodimer (in green) aligned with the mutant pre-pro-TGFβ1 homodimer (in blue), created using AlphaFold2 and PyMOL. The p. Leu11_Leu16 duplication is highlighted in magenta within the signal peptide at the N-terminus of the mutant homodimer. (**D**) Schematic of wild and mutant pre-pro-TGFβ1 proteins. Please note AlphaFold2 is not validated for predicting the structural effects of variants. This model is shown only to illustrate the position of the variant within the protein. (**E**) Sanger sequencing traces of the *TGFB1* gene in the proband and both parents, shown against a synthesized reference trace using Mutation Surveyor. The proband carries a heterozygous c.31_48dup duplication (highlighted by red arrows), visible as overlapping peaks following nucleotide c.48. This duplication was not present in either parent

A diagnosis of juvenile Paget’s disease was made, based on radiographic appearances and hearing loss; however, sequencing of known monogenic causes did not identify any causative variants. Bisphosphonates were not started due to the elevated BMD.

He re-presented in 2020 with worsening bone and joint pain, exacerbated by walking, alongside a deterioration in his hearing. ALP was now elevated at 411 U/l. Audiogram showed progressive severe bilateral sensory hearing loss; cranial computed tomography showed diffuse skull thickening with almost complete obliteration of both internal auditory canals.

His hearing aids were optimized. Alendronate was commenced, leading to almost complete resolution of musculoskeletal symptoms within 6 weeks, and partial suppression of CTX (1.03 µg/l) at 9 months (P1NP not tested).

Recurrence of bone and joint pains in 2022 prompted a switch to IV zoledronate; however, he reported worsening bone pain and hearing loss within a week of the first dose, persisting for 2 months. CTX was also high (1.2 µg/l) [no pre-dose measurement]. Alendronate was restarted and musculokeletal symptoms improved within 12 weeks.

In 2024, he presented with increased muscle weakness, unsteadiness, and fatigue. Skeletal dysplasia whole genome sequence (WGS) sequencing panel (R104 version 4.0, https://nhsgms-panelapp.genomicsengland.co.uk/) was initially reported as showing no causative variants. However, upon review of additional clinical information and variants of uncertain significance (VUS), a single heterozygous c.31_48 inframe duplication in exon 1 of *TGFB1* was identified as a candidate variant. This was predicted to lead to an in-frame duplication of 6 residues p.(Leu11_Leu16dup) in TGFβ1. Protein modelling using AlphaFold2 [2] demonstrated that the variant was located within the N-terminal signal peptide of the TGFβ1 pro-protein (p.Leu11_Leu16dup) (Fig. 1C, D) [2]. Subsequent family testing confirmed the variant to be *de novo.* His clinical history, biochemistry and imaging were reassessed and considered consistent with CED.

TGFβ1 is a multifunctional protein with key roles in bone and muscle physiology; perturbations in the TGFβ1 signalling pathway are associated with multiple skeletal dysplasias (e.g. osteogenesis imperfecta type IV (OMIM 166220), acromicric dysplasia (OMIM 102370) and Loeys-Dietz syndrome types 1–5. Most (>80%) individuals with CED type 1 have activating missense variants in exon 4, affecting the latency-associated peptide of pro-TGFβ1 [1]. Two duplications of 3 and 4 leucine residues within the signal peptide have previously been described [3, 4]. Our case has the largest duplication reported of 6 residues (LLLPLL), extending the hydrophobic region within the signal peptide from 14 to 20 amino acids. This is likely to impact proper cleavage of the signal peptide and reduce pro-TGFβ1 secretion, leading to intracellular accumulation. This has been shown to activate TGFβ1 signalling *in vitro*, possibly via alternative pathways [3].

Management of CED is supportive, with inconsistent evidence on the symptomatic benefit of corticosteroids, non-steroidal anti-inflammatory drugs, losartan and/or bisphosphonates [5–8]. Unexpectedly, our patient experienced discordant effects on his symptoms with alendronate versus zoledronate. This was unexpected, given the greater potency of zoledronate, and has not been observed previously. Speculatively, rapid bone turnover in CED may affect skeletal retention of bisphosphonates, with differential effects from gradual administration versus infusion. Bone turnover marker measurements were limited here; monitoring may help to guide and interpret treatment responses.

Our case highlights the importance of WGS analysis in diagnostically challenging cases. Furthermore, trialling different bisphosphonates in individuals with CED may aid symptom management.

## Data Availability

Anonymised data are available upon reasonable request by any qualified researchers who engage in rigorous, independent scientific research, and will be provided following review and approval of a research proposal and Statistical Analysis Plan (SAP) and execution of a Data Sharing Agreement (DSA). The data are not publicly available due to privacy or ethical restrictions.

## References

[1] Van Hul W , Boudin E, Vanhoenacker FM, Mortier G. Camurati–Engelmann disease. Calcified tissue international. Review 2019;104:554–60. 10.1007/s00223-019-00532-1 30721323

[2] Jumper J , Evans R, Pritzel A et al Highly accurate protein structure prediction with AlphaFold. Nature 2021;596:583–9. 34265844 10.1038/s41586-021-03819-2 PMC8371605

[3] Janssens K , Ten Dijke P, Ralston SH, Bergmann C, Van Hul W. Transforming growth factor-β1 mutations in Camurati-Engelmann disease lead to increased signaling by altering either activation or secretion of the mutant protein. J Biol Chem 2003;278:7718–24. 12493741 10.1074/jbc.M208857200

[4] Whyte MP , Totty WG, Novack DV et al Camurati-engelmann disease: unique variant featuring a novel mutation in TGFβ1 encoding transforming growth factor beta 1 and a missense change in TNFSF11 encoding RANK ligand. J Bone Miner Res 2011;26:920–33. 21541994 10.1002/jbmr.283 PMC3179308

[5] Janssens K , Vanhoenacker F, Bonduelle M et al Camurati-Engelmann disease: review of the clinical, radiological, and molecular data of 24 families and implications for diagnosis and treatment. J Med Genet 2006;43:1–11. 15894597 10.1136/jmg.2005.033522 PMC2564495

[6] Combier A , Palazzo E, Forien M et al Failure of conventional treatment and losartan in Camurati-Engelmann disease: a case report. Joint Bone Spine 2018;85:649–50. 29452301 10.1016/j.jbspin.2018.01.015

[7] Baroncelli GI , Ferretti E, Pini CM et al Significant improvement of clinical symptoms, bone lesions, and bone turnover after long-term zoledronic acid treatment in patients with a severe form of Camurati-Engelmann disease. Mol Syndromol 2017;8:294–302. 29230158 10.1159/000479859 PMC5701277

[8] Klemm P , Aykara I, Lange U. Camurati-Engelmann disease: a case-based review about an ultrarare bone dysplasia. Eur J Rheumatol 2023;10:34–8. 36880809 10.5152/eurjrheum.2023.21115 PMC10152113

